# STAT3 Inhibitor ODZ10117 Suppresses Glioblastoma Malignancy and Prolongs Survival in a Glioblastoma Xenograft Model

**DOI:** 10.3390/cells9030722

**Published:** 2020-03-15

**Authors:** Byung-Hak Kim, Haeri Lee, Cheol Gyu Park, Ae Jin Jeong, Song-Hee Lee, Kum Hee Noh, Jong Bae Park, Chung-Gi Lee, Sun Ha Paek, Hyunggee Kim, Sang-Kyu Ye

**Affiliations:** 1 Department of Pharmacology, Seoul National University College of Medicine, Seoul 03080, Korea; protein0826@snu.ac.kr (B.-H.K.); hrlee519@snu.ac.kr (H.L.); lovej89@snu.ac.kr (A.J.J.); 24happy92@snu.ac.kr (S.-H.L.); nkhys1209@snu.ac.kr (K.H.N.); 2 Biomedical Science Project (BK21PLUS), Seoul National University College of Medicine, Seoul 03080, Korea; 3 CYTUS H&B Corporation, Cheongju 28159, Korea; cglee2020@gmail.com; 4 Department of Biotechnology, School of Life Sciences and Biotechnology, Korea University, Seoul 02841, Korea; pcpc89@korea.ac.kr (C.G.P.); hg-kim@korea.ac.kr (H.K.); 5 Department of System Cancer Science, Graduate School of Cancer Science and Policy, National Cancer Center, Goyang 10408, Korea; jbp@ncc.re.kr; 6 Department of Neurosurgery, Seoul National University College of Medicine, Seoul 03080, Korea; paeksh@snu.ac.kr; 7 Cancer Research Institute, Seoul National University College of Medicine, Seoul 03080, Korea; 8 Ischemic/Hypoxic Disease Institute, Seoul National University College of Medicine, Seoul 03080, Korea; 9 Neuro-Immune Information Storage Network Research Center, Seoul National University College of Medicine, Seoul 03080, Korea

**Keywords:** glioblastoma, glioma stem cell (GSC), ODZ10117, STAT3, targeted therapy

## Abstract

Constitutively activated STAT3 plays an essential role in the initiation, progression, maintenance, malignancy, and drug resistance of cancer, including glioblastoma, suggesting that STAT3 is a potential therapeutic target for cancer therapy. We recently identified ODZ10117 as a small molecule inhibitor of STAT3 and suggested that it may have an effective therapeutic utility for the STAT3-targeted cancer therapy. Here, we demonstrated the therapeutic efficacy of ODZ10117 in glioblastoma by targeting STAT3. ODZ10117 inhibited migration and invasion and induced apoptotic cell death by targeting STAT3 in glioblastoma cells and patient-derived primary glioblastoma cells. In addition, ODZ10117 suppressed stem cell properties in glioma stem cells (GSCs). Finally, the administration of ODZ10117 showed significant therapeutic efficacy in mouse xenograft models of GSCs and glioblastoma cells. Collectively, ODZ10117 is a promising therapeutic candidate for glioblastoma by targeting STAT3.

## 1. Introduction

Glioblastoma is the most frequent and most aggressive occurring type of primary brain tumor in adults. It is positively correlated with a deadly disease with extremely poor prognosis, early clinical deterioration, and high mortality rate. Patients with glioblastoma show a median overall survival of less than 15 months and a 5-year survival rate of less than 5% despite surgical intervention with radiotherapy and chemotherapy [1,2]. Although extensive studies and advances in modern medicine during past decades have occurred, glioblastoma remains difficult to treat with a very dismal prognosis in patients, because few mechanisms underlying its tumor malignancy have been identified. Therefore, it is necessary to develop novel therapeutic strategies to improve the survival rate and prognosis of glioblastoma. Recently, the degree of signal transducer and activator of transcription 3 (STAT3) activity has emerged as an important biomarker in targeted therapy of cancer patients, including glioblastoma patients [3,4], suggesting that STAT3 inhibitors may be an essential strategy for the treatment of glioblastoma.

STAT3 is a transcription regulator involved in many intracellular functions, including cell proliferation, differentiation, survival, angiogenesis, and immune response. It can be activated directly or indirectly by receptor-associated and non-receptor-associated tyrosine kinases such as Janus kinases (JAKs), Src family kinases and Bcr-Abl kinase [5,6]. Accumulated evidence demonstrated that constitutively activated STAT3 is observed in various types of tumor-derived cell lines and tumor tissues, including diverse solid and hematologic cancers [7,8,9,10,11]. Constitutively activated STAT3 is closely related to oncogenic signaling, recurrence and drug resistance that increases cancer aggressiveness and malignancy by promoting the survival, proliferation, invasion, and metastasis of cancer cells and maintenance of cancer stem cell (CSC) properties in a wide range of cancers in the inflammation-associated tumor microenvironment [5,6]. Therefore, targeting STAT3 is a potentially valuable strategy for cancer therapy, and several STAT3 inhibitors have been developed, some of which are under clinical trials [12,13,14]. The inhibitors are commonly based on direct targeting of STAT3 or indirect targeting of the upstream regulators of STAT3 signaling.

We previously reported that oxadiazole-based small molecule 3-(2,4-dichloro-phenoxymethyl)-5-trichloromethyl-[1,2,4]oxadiazole (ODZ10117) is a direct inhibitor of STAT3 by targeting the SH2 domain of STAT3 [15]. In the present study, we demonstrated the pharmacological effect of ODZ10117 in both in vitro and in vivo models of glioblastoma and glioblastoma cancer stem cells (GSCs).

## 2. Materials and Methods

### 2.1. Preparation of Primary Cell Lines

Human glioblastoma tissue samples were obtained from the patients with glioblastoma who visited the Department of Neurosurgery, Seoul National University College of Medicine. All the patients participating in this study provided informed consent before the surgical procedure at the Seoul National University Hospital. The study was approved by the Institutional Review Board protocols from Seoul National University College of Medicine (H-0507-509-153) in accordance with the Declaration of Helsinki. The primary glioblastoma cell lines were prepared from the tumor tissue samples of glioblastoma patients code-named with GBM12, 13, 14, 28, 30, and 37, and maintained in DMEM (SH30243.01, HyClone, Carlsbad, CA, USA) supplemented with 10% FBS (16000044, Gibco, Carlsbad, CA, USA) and 1% penicillin/streptomycin (15070063, Gibco).

### 2.2. Cell Lines

Human glioblastoma cell lines A172 (CRL-1620), Hs683 (HTB-138), T98G (CRL-1690), U87-MG (HTB-14), U118-MG (HTB-15), U138-MG (HTB-16), and U373-MG (HTB-17) were obtained from the American Type Culture Collection (Manassas, VA, USA), and U251-MG (300385) cells were obtained from the CLS Cell Lines Service (Eppelheim, Germany). All the cells were maintained in DMEM supplemented with 10% FBS and 1% penicillin/streptomycin. GSC lines 19, 84, and 528 were obtained from Dr. Ichiro Nakano (The Ohio State University, Columbus, OH, USA) and maintained in DMEM/F12 (SH30023.01, HyClone) supplemented with 0.04% modified B27 (17504044, Invitrogen, Carlsbad, CA, USA), 1% L-glutamine (25030081, Invitrogen), 20 ng/mL basic fibroblast growth factor (100-18B, PeproTech, Rocky Hill, NJ, USA) and 20 ng/mL epidermal growth factor (GMP100-15, PeproTech). All the cells were maintained at 37 °C in a 5% CO_2_ humidified incubator.

### 2.3. Reagents and Antibodies

The known STAT3 inhibitors S3I-201 (SML0330) [16], STA-21 (SML2161) [17], and nifuroxazide (481984) [18], and the pan-JAK inhibitor AG-490 (T3434) [19] were purchased from Sigma-Aldrich (St. Louis, MO, USA). An inhibitor of STAT3 and cancer stemness, napabucasin (HY-13919) [20] and recombinant human interleukin 6 (IL-6, 200-06) were obtained from MedChem Express (Monmouth Junction, NJ, USA) and PeproTech, respectively. All of the other chemicals used were analytical grade and purchased from Sigma-Aldrich unless otherwise noted.

Antibodies against phospho-STAT3 (9145), NESTIN (4760), SOX2 (3579), NANOG (4903), OCT4 (2840), PARP (9542), caspase-3 (9662), and active caspase 3 (9661), and Bcl-_X_L (2762) were obtained from Cell Signaling Technology (Danvers, MA, USA). Antibodies against fibronectin (ab2413), integrin αV (ab179475), MMP-9 (ab38898), CXCR4 (ab124824), TWIST (ab49254), and Ki-67 (ab15580) were obtained from Abcam (Cambridge, MA, USA). Antibodies specific for STAT3 (sc-482), CD133 (orb10288), pro/active MMP-2 (MAB902 and NB200-193), and GAPDH (AbC-2003) were obtained from Santa Cruz Biotechnology (Santa Cruz, CA, USA), Biorbyt (St. Louis, MO, USA), R&D systems (Minneapolis, MN, USA), Novus Biologicals (Centennial, CO, USA), and Abclone (Seoul, Korea), respectively.

### 2.4. Western Blot Analysis

Whole-cell lysates were prepared using a lysis buffer containing 50 mM Tris-HCl (pH 7.4), 350 mM NaCl, 1% Triton X-100, 0.5% Nonidet P-40, 10% glycerol, 0.1% SDS, 1 mM EDTA, 1 mM EGTA, 1 mM Na_3_VO_4_, 1 mM phenylmethylsulphonyl fluoride, and protease and phosphatase inhibitor cocktails (78440, Thermo Scientific, Rockford, IL, USA). Protein samples were separated by SDS-PAGE, transferred to nitrocellulose membrane (Pall Corporation, Port Washington, NY, USA), and performed Western blotting with appropriate antibodies. All primary antibodies except STAT3 and GAPDH were diluted at 1:1000, while STAT3 and GAPDH antibodies were diluted at 1:2000 and 1:5000, respectively.

### 2.5. RNA Extraction and Quantitative Real-Time PCR

Total RNA was isolated using a TRIzol Reagent (Invitrogen) and cDNA was synthesized using a ReverTra Ace^®^ qPCR RT Kit (Toyobo, Osaka Japan). Quantitative real-time PCR (qPCR) was performed using a QuantiFast SYBR Green PCR master mix (Qiagen, Valencia, CA, USA) with an Applied Biosystems 7300 (Life Technologies, Carlsbad, CA, USA). The data were analyzed by comparative C_t_ quantification and the value for each sample was normalized to the value for the housekeeping *GAPDH* gene. The primer pairs used in this experiment were BCL-2 (QT00025011), BCL-XL (QT00236712), SURVIVIN (QT01679664), MMP-2 (QT00088396), MMP-9 (QT00040040), FN (QT00038024), ITGAV (QT00051891), VIMENTIN (QT00095795), TWIST (QT00011956), CCND1 (QT00495285), MYC1 (QT00035406), NESTIN (QT01015301), SOX2 (QT00237601), NANOG (QT01844808), OCT4 (QT00210840), CD133 (QT00075586), and GAPDH (QT0007924) were obtained from Qiagen.

### 2.6. Cell Viability Assay

Cells were seeded at 10,000 cells per well in 96-well plates and incubated in culture medium until 70–80% confluence. The cells were further incubated for 24 h with either vehicle alone or various concentrations of ODZ10117. Cell viability was measured at 450 nm using microplate reader (Molecular Devices, Sunnyvale, USA) after being further incubated for 2–4 h at 37 °C following the addition with EZ-CyTox Enhanced Cell Viability Assay Reagent (Daeil Lab Service, Seoul, Korea).

### 2.7. Immunofluorescence Staining

Cells grown in lysine-coated 24-well plates were fixed for 45 min at room temperature in 3% paraformaldehyde in PBS and permeabilized for 10 min with 0.1% Triton X-100 in PBS. The plates were blocked for 20 min with 3% BSA in PBS and incubated with tyrosine phosphorylated STAT3 (pY^705^-STAT3) antibody at 4 °C overnight. After washing with PBS, the dishes were incubated with fluorescein isothiocyanate (FITC)-conjugated secondary antibody at room temperature for 2 h. Nuclei were counterstained with 4′,6-diamidino-2-phenylindole (DAPI, D8417, Sigma-Aldrich) and images were captured using a Zeiss Axiovert 200 inverted fluorescence microscope (Oberkochen, Germany) with an LSM 510 META system (ZEN 2011). pY^705^-STAT3 antibody was used at 1:200 dilution.

### 2.8. Tissue Staining and Immunohistochemistry

Tissue samples were fixed with 4% paraformaldehyde in 0.5 M phosphate buffer and embedded in paraffin. The paraffin blocks were cut in 4-μm-thick sections, mounted on glass slides, dewaxed, rehydrated with grade ethanol, and stained with hematoxylin and eosin (H&E, HT100132, Sigma Aldrich and S3309, Dako, Carpinteria, CA, USA). To perform immunohistochemical analysis, rehydrated slide sections were unmasked with 10 mM sodium citrate buffer, quenched endogenous peroxidase for 20 min in 3% hydrogen peroxide, blocked for 30 min in PBS containing 10% goat serum, and incubated at 4 °C for overnight with appropriate primary antibodies with 1:100 dilution. The sections were incubated with biotinylated secondary antibody (anti-rabbit for BA-1000, anti-mouse for BA-9200 and anti-goat for BA-5000, Vector Labs, Burlingame, CA, USA) compatible with the primary antibody for 30 min, subsequently incubated with streptavidin-HRP (550946, BD Pharmingen, San Jose, CA, USA) for 40 min, and stained with 3,3-diaminobenzidine (D22187, Invitrogen). Digital images were obtained using the LAS Microscope Software (Leica Microsystems, Wetzlar, Germany).

### 2.9. Flow Cytometry

Dissociated single cells of GSCs were washed with PBS and fixed with 4% paraformaldehyde at 4 °C for 10 min in the dark. Fixed cells were washed twice in ice-cold FACS buffer (00-4222-26, eBioScience, Carlsbad, CA, USA) containing 3% BSA in PBS and incubated with phycoerythrin (PE)-conjugated CD133 antibody (130-113-108, 1:20 dilution, Miltenyi Biotec, Sunnyvale, CA, USA). After 1 h incubation at 4 °C, the cells were washed twice with PBS and incubated with PE-conjugated avidin (554061, BD Pharmingen). To analyze cell cycle and apoptotic cell population, cells were fixed with 70% ice-cold ethanol, washed with PBS, incubated with RNase (50 μg, 10109134001, Sigma Aldrich) at 37 °C for 1 h, and stained with propidium iodide (PI, 20 μg, 556463, BD Biosciences, San Jose, CA, USA) at 4 °C in the dark. For Annexin V staining, Annexin V binding buffer (422201, BioLegend, San Diego, CA, USA) containing fluorescein isothiocyanate (FITC) conjugated with anti-Annexin V antibody (640906, 1:50 dilution, BioLegend) was used as manufacturer’s protocol. Stained cells were counted with flow cytometry using the BD LSRFortessa^TM^ cell analyzer (BD Biosciences).

### 2.10. Wound Healing and Invasion Assays

To conduct wound healing assay, cells were seeded into 12-well plates and then incubated over 90% confluence. The plate was scratched with pipette tips and washed with PBS. Cells were incubated for 24 h with fresh DMEM medium containing either vehicle alone or ODZ10117. Digital images were obtained using the Leica Application Suite (LAS) Microscope Software (Leica Microsystems).

Invasion assay was performed using a Boyden chamber system (Neuro Probe, Gaithersburg, MD, USA). Growth factor reduced Matrigel (354230, BD Matrigel™, BD Biosciences) was diluted with serum free media with ratio of 1:3. Diluted Matrigel was transferred into 24-transwell (BD 24-well insert, 8 μm pore transparent PET filter) and incubated at least for 4 to 5 h for gelling at 37 °C. Cells in 100 μL DMEM containing 1% FBS were seeded in the upper chamber and incubated for 24 h in the presence of either vehicle alone or ODZ10117. The lower chamber was filled with 500 μL of 10% DMEM containing fibronectin (5 μg/mL, ECM001, Sigma-Aldrich). Matrigel containing upper chamber was rinsed with PBS, fixed, stained with Diff-Quik solution (Sysmex Corporation, Kobe, Japan), and subsequently rinsed with distilled water. The migrated cells were captured using the LAS Microscope Software (Leica Microsystems).

### 2.11. In vitro Limiting Dilution and Sphere-Forming Assays

Sequentially decreasing numbers of GSCs were seeded into 96-well plates and incubated for 2 weeks. Colonies were counted and photographed with microscope (Olympus, Tokyo, Japan). Stem cell frequency was calculated using the extreme limiting dilution analysis (ELDA) software [21]. To determine sphere-forming capacity, dissociated single cells were seeded at 10,000 cells per well into 24-well plates and incubated for 5 days in the presence of vehicle alone, ODZ10117, or each of the known STAT3 inhibitors. Sphere formation was captured using a microscope (Olympus).

### 2.12. In vivo xenograft models

To generate an orthotopic xenograft model, GSC528 cells (5 × 10^4^ cells in 3 µL of PBS) were stereotactically injected into the right striatum of 6-week-old BALB/c nu/nu nude mice (coordinates relative to the bregma: medial-lateral +2 mm, and dorsal-ventral −3 mm). After 14 days, mice were randomly divided into three groups (*n* = 7) receiving an intraperitoneally injection of ODZ10117 (0.1 mg/kg or 1 mg/kg) in solvent (DMSO:corn oil, 1:9) or equal volume of solvent only (control). Mice were injected six times per week and body weight was measured every day. Animals were monitored daily after treatment for the manifestation of any pathological signs. To compare the tumor histology, all mice were sacrificed at the same time when one mouse initially exhibited neurological symptoms. To generate a subcutaneous tumor xenograft model, U87-MG cells (2.5 × 10^6^ cells in 100 μL of 25% Matrigel containing PBS) were subcutaneously injected into the flank of 6-week-old BALB/c nu/nu nude mice**.** After 14 days, mice were randomly divided into two groups (*n* = 6). Each group was treated with vehicle alone or ODZ10117 with a 2-day interval for 2 weeks. The tumor sizes were measured by a caliper ruler with a 2-day interval, and the tumor volumes were calculated using *a* × *b*^2^/2 (where *a* is the width at the widest point of the tumor and *b* is the width perpendicular to *a*).

### 2.13. Statistics

Each experiment was performed independently at least twice, and the results are represented as the mean ± standard error of mean (SEM), unless otherwise indicated. Statistical analyses were performed with GraphPad Prism 5.0 (GraphPad Software, San Diego, CA, USA). The significance was determined using a two-tailed Student’s *t*-test and values of *p* < 0.05 were considered statistically significant. Survival analysis was conducted by the Kaplan-Meier method with the log-rank test.

## 3. Results

### 3.1. STAT3 Hyperactivation Increases Tumor Malignancy and Decreases Survival in Glioblastoma Patients

**Figure 1:**
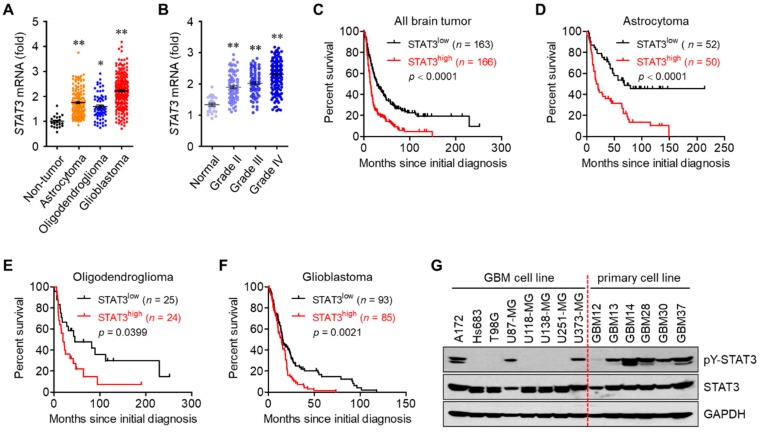
STAT3 hyperactivation is associated with tumor malignancy and survival in glioblastoma patients. (**A**,**B**) The mRNA level of STAT3 in non-tumor (normal) controls and different types of glioma tissues (**A**) and patients with gliomas of various grades (**B**). Results were analyzed from TCGA database. * *p* < 0.05 and ** *p* < 0.005. (**C**–**F**) Kaplan-Meier survival curves for patients with all brain tumors (**C**), astrocytomas (**D**), oligodendrogliomas (**C**), and glioblastomas (**F**). Data from the TCGA database. (**G**) Western blot results of glioblastoma and primary glioblastoma cell lines established from the patients.

Glioblastoma represents various features of tumor malignancy and high resistance to current therapeutic approaches such as surgery combined with radiochemotherapy [1,2,4]. To investigate the negative roles of STAT3 in brain tumors, we analyzed the database of The Cancer Genomic Atlas (TCGA) Research Network (http://www.betastasis.com/glioma/). The mRNA level of STAT3 was higher in glioblastoma compared to non-tumor, astrocytoma, and oligodendroglioma (Figure 1A), and was positively correlated with the grade of brain tumor (Figure 1B). In addition, the survival rate was shorter with the higher mRNA level of STAT3 in patients with all brain tumors, astrocytoma, oligodendroglioma, and glioblastoma (Figure 1C–F). We further analyzed the level of active STAT3 (phosphorylated at tyrosine 705 residue) in glioblastoma and primary glioblastoma cell lines. In particular, the level of active STAT3 was relatively higher in five patients, and these patients had a shorter survival rate than the remaining patient code-named with GBM12 (Figure 1G). The overall mean survival rates were 433 ± 276 days in the six patients, 340 ± 173 days in patients with higher levels of active STAT3, and 899 days in patients called GBM12, with lower levels of active STAT3. These results indicate that STAT3 is closely correlated with tumor malignancy and decreases survival in glioblastoma patients.

### 3.2. ODZ10117 Inhibits STAT3 Activation in Glioblastoma Cells

**Figure 2:**
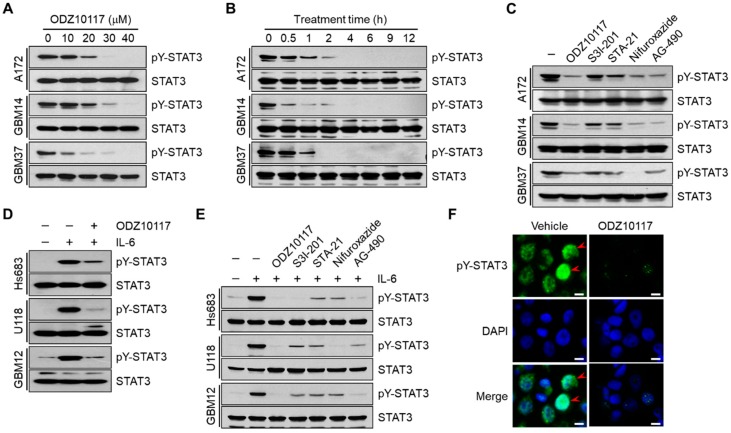
ODZ10117 inhibits tyrosine phosphorylation of STAT3 in glioblastoma cells. (**A**–**C**) Western blot analyses were performed from the glioblastoma cells incubated for 12 h with ODZ10117 in a concentration-dependent manner (**A**), 40 μM ODZ10117 in a time-dependent manner (**B**) or for 12 h with ODZ10117 (40 μM) and the known STAT3 inhibitor S3I-201 (100 μM), STA-21 (100 μM), nifuroxazide (100 μM), or AG-490 (150 μM) (**C**). (**D**,**E**) ODZ10117 and the known STAT3 inhibitors were treated for 12 h and then stimulated with IL-6 (20 ng/mL) for 10 min. (**F**) Immunofluorescence staining was performed using an anti-pY^705^-STAT3 antibody (green) in GBM14 cells incubated for 24 h with vehicle (0.1% DMSO) alone or ODZ10117 (40 μM). Nuclei were stained with DAPI (blue). Arrowheads indicate tyrosine-phosphorylated STAT3 in the nucleus. Scale bars: 20 μm

Glioblastoma is the most incurable type of cancer with few therapeutic options, and STAT3 is a promising therapeutic target for glioblastoma [4,22]. ODZ10117 was previously reported as a direct inhibitor of STAT3 by targeting the SH2 domain of STAT3 [15]. Therefore, we investigated whether ODZ10117 may affect STAT3 activation in glioblastoma and primary glioblastoma cells. Tyrosine phosphorylated STAT3 was effectively inhibited following incubation with >20 μM ODZ10117 for over 12 h (Figure 2A) and with 40 μM ODZ10117 over for 1 h (Figure 2B, and Figure S1A in Supplementary Materials) in glioblastoma and primary glioblastoma cell lines. We further compared the inhibitory effect of ODZ10117 with known STAT3 inhibitors S3I-201 [16], STA-21 [17], and nifuroxazide [18] together with the pan-JAK inhibitor AG-490 [19]. The inhibitory efficacy of 40 μM ODZ10117 was compared with 100 μM nifuroxazide or 150 μM AG-490. However, 100 μM S3I-201 and 100 μM STA-21 had weaker inhibitory activity than ODZ10117 in the cell lines (Figure 2C and Figure S1B). In addition, ODZ10117 also effectively inhibited IL-6-induced STAT3 activation in glioblastoma and primary glioblastoma cell lines expressing lower level of active-STAT3 (Figure 2D and Figure S1C), and the inhibitory efficacy of ODZ10117 with the known STAT3 inhibitors and AG-490 was similar to that of glioblastoma and primary glioblastoma cell lines expressing higher levels of active STAT3 (Figure 2E and Figure S1D). Furthermore, immunofluorescence staining results showed that ODZ10117 decreased the nuclear translocation of active STAT3 compared to that in the vehicle-treated control group (Figure 2F). The abovementioned results indicate that ODZ10117 is a potential STAT3 inhibitor in glioblastoma and the effect is greater than that of the known STAT3 inhibitors such as S3I-201, STA-21, nifuroxazide, and AG-490.

### 3.3. ODZ10117 Reduces the Migration and Invasion of Glioblastoma Cells

**Figure 3:**
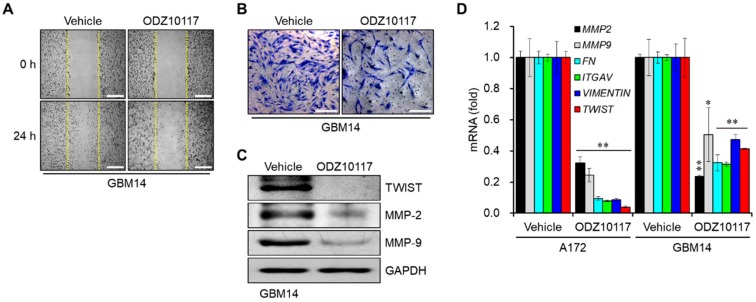
ODZ10117 decreases the migration and invasion of glioblastoma cells. (**A**,**B**) Wound healing (**A**) and Matrigel invasion (**B**) assays of cells from GBM14 incubated for 24 h with vehicle (0.1% DMSO) alone or ODZ10117 (40 μM). Scale bars: 200 μm (**A**), 500 μm (**B**). The images were visualized by phase-contrast microscopy (magnification = 200×). (**C**,**D**) Western blot (**C**) and qPCR (**D**) analyses were performed in glioblastoma cells incubated for 24 h with vehicle (0.1% DMSO) alone or ODZ10117 (40 μM). GAPDH served as the loading control and data represent the mean ± SEM of three independent experiments. * *p* < 0.05 and ** *p* < 0.005.

The migration and invasion of cancer cells into the bloodstream and surrounding tissues are critical steps in cancer metastasis, and the transcription of target genes associated with these processes is regulated by STAT3 in the tumor microenvironment [23]. In glioblastoma, extracranial metastasis is rare, however, migration and invasion of cancer cells are significant features. The results of wound healing assay revealed that the migration of glioblastoma cells was regulated by STAT3, which was decreased in the presence of ODZ10117 compared to the vehicle-treated control group in primary glioblastoma cell line GBM14 (Figure 3A).

We next conducted in vitro Matrigel invasion assay to determine the effect of ODZ10117 in the invasiveness of glioblastoma cells. The lower chamber was filled with culture medium containing 5 ng/mL fibronectin, and primary glioblastoma cells were maintained in the upper chamber for 24 h. The inhibition of STAT3 activity by ODZ10117 decreased the invasiveness of GBM14 cells (Figure 3B). The protein levels of TWIST, MMP-2, and MMP-9, targets of STAT3 associated with migration and invasion, were decreased by ODZ10117 compared to the vehicle-treated control group (Figure 3C). Consistently, the mRNA levels of *MMP-2*, *MMP-9*, *FN*, *ITGAV*, *VIMENTIN,* and *TWIST* were effectively suppressed by ODZ10117 in glioblastoma and primary glioblastoma cell lines (Figure 3D), which suggests that ODZ10117 may suppress cancer metastasis by inhibiting the expression of target genes associated with STAT3-dependent migration and invasion in glioblastoma.

### 3.4. ODZ10117 Decreases the Viability of Glioblastoma Cells by Inducing Apoptosis

**Figure 4:**
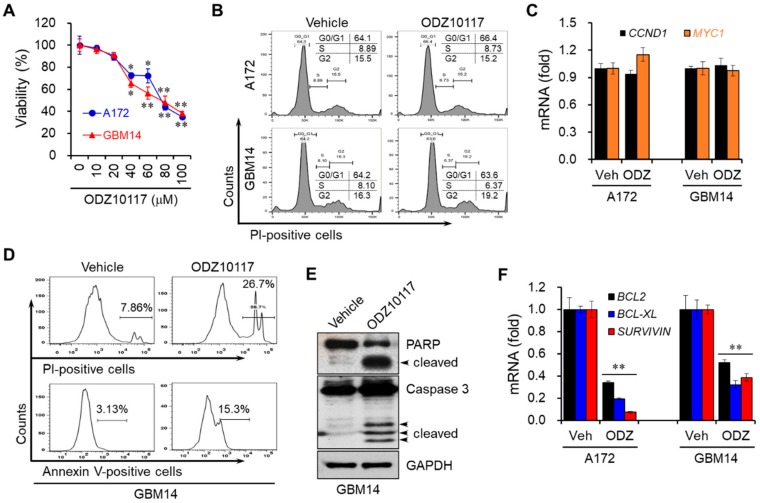
ODZ10117 induces apoptotic cell death in glioblastoma cells. (**A**) Cells were incubated with various concentrations of ODZ10117 for 24 h and their viability was measured. * *p* < 0.05 and ** *p* < 0.005. (**B**,**C**) Cells were incubated for 24 h with vehicle (0.1% DMSO) or ODZ10117 (40 μM), and proliferation and proliferation markers were determined by FACS (**B**) and qPCR (**C**) analyses. (**D**–**F**) Cells were incubated for 24 h with vehicle (0.1% DMSO) alone or ODZ10117 (40 μM) and subjected to FACS (**D**), Western blot (**E**), and qPCR (**F**) analyses. GAPDH served as the loading control and data represent the mean ± SEM of three independent experiments. ** *p* < 0.005.

We further determined whether ODZ10117 could affect the viability and proliferation of glioblastoma cells because STAT3 regulates the survival and proliferation of cancer cells [24]. ODZ10117 decreased the viability of glioblastoma and primary glioblastoma cell lines in a concentration-dependent manner (Figure 4A). However, the results of FACS analyses revealed that the proliferation of glioblastoma cells was not affected by ODZ10117 (Figure 4B). Consistently, it did not alter the mRNA levels of cyclin D1 (*CCND1*) and *MYC1* pertinent to cell cycle progression (Figure 4C), suggesting that ODZ10117 affects the viability of glioblastoma cells, but not proliferation.

To determine whether the decreased viability of glioblastoma cells by ODZ10117 resulted from apoptotic cell death, we performed FACS analyses, followed by staining with propidium iodide (PI) and annexin V in GBM14 cells. The population of dead cells was increased more than threefold, and among the dead cells, apoptotic cell death was increased about fivefold by ODZ10117 compared to that in the vehicle-treated control group (Figure 4D). ODZ10117 increased the cleaved forms of both PARP and caspase-3 in the cells, which are the hallmarks of apoptosis (Figure 4E). In addition, ODZ10117 downregulated the mRNA levels of *BCL-2*, *BCL-XL*, and *SURVIVIN* (Figure 4F), anti-apoptotic genes regulated by STAT3 [5,6]. These results indicate that ODZ10117 decreases the survival of glioblastoma cells by inducing apoptotic cell death via activation of the apoptotic proteins and inhibition of the expression of anti-apoptotic genes.

### 3.5. ODZ10117 Decreases Stem Cell Properties by Inhibiting STAT3 in Glioblastoma Stem Cells

CSC properties and metastatic capabilities are implicated with tumor malignancy, recurrence and drug resistance in various types of cancer, which are closely associated with STAT3 signaling in the inflammatory tumor microenvironment [25]. To investigate the roles of STAT3 on CSC properties and metastatic capabilities in glioblastoma patients, we analyzed the database of TCGA Research Network. The data revealed that the mRNA level of STAT3 was positively correlated with stem cell-related genes such as *CD133*, *NESTIN*, and *SOX2* and mesenchymal (MES)-associated genes such as *FN* and *ITGAV* (Figure S2A).

Glioblastoma is classified into four subtypes according to the gene expression pattern: proneural (PN), neural (N), classical (CL), and MES [26,27]. Analyses of the TCGA datasets revealed that the mRNA levels of stem cell-related and MES-associated genes were significantly elevated in all subtypes of glioblastoma patients compared to normal brain tissue (Figure S2B) and were associated with a decreased survival rate of glioblastoma patients (Figure S2C), suggesting the important role of STAT3 in tumor malignancy, recurrence and drug resistance by maintaining CSC properties and enhancing metastatic capabilities in glioblastoma.

**Figure 5:**
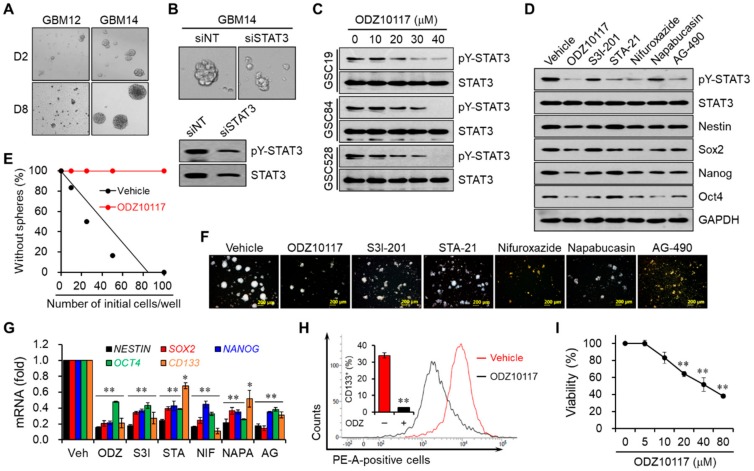
ODZ10117 suppresses stemness features in glioblastoma and GSCs. (**A**,**B**) Sphere-forming assay was performed in GBM12 and GBM14 cells following incubation for 2 or 8 days (**A**), or in GBM14 cells transfected with the control or STAT3 siRNA following incubation for 8 days (**B**) under stem cell culture conditions. (**C**,**D**) Western blot analysis was performed for STAT3 activation and/or stem cell markers in GSCs incubated for 24 h with ODZ10117 (**C**) and in GSC528 cells incubated for 24 h with ODZ10117 and the known STAT3 inhibitors (**D**). GAPDH served as the loading control. ODZ10117 (40 μM), S3I-201 (100 μM), STA-21 (100 μM), nifuroxazide (100 μM), napabucasin (4 μM), and AG-490 (150 μM). (**E**,**F**) In vitro limiting dilution (**E**, *n* = 12) and sphere-forming (**F**) assays were performed in GSC528 cells incubated for 5 days with vehicle (0.1% DMSO) alone, ODZ10117 and the known STAT3 inhibitors. ODZ10117 (40 μM), S3I-201 (100 μM), STA-21 (100 μM), nifuroxazide (100 μM), napabucasin (4 μM), and AG-490 (150 μM). Scale bars: 200 μm. (**G**,**H**) The mRNA levels of the stem cell markers and CD133-positive cell population were determined by qPCR (**G**) and FACS (**H**) analyses in GSC528 cells incubated for 24 h with vehicle (0.1% DMSO) alone, ODZ10117 and the known STAT3 inhibitors. ODZ10117 (ODZ, 40 μM), S3I-201 (S3I, 100 μM), STA-21 (STA, 100 μM), nifuroxazide (NIF, 100 μM), napabucasin (NAPA, 4 μM), and AG-490 (AG, 150 μM). (**I**) Viability was determined in GSC528 cells incubated for 24 h with various concentrations of ODZ10117. Data represent the mean ± SEM of three independent experiments. * *p* < 0.05 and ** *p* < 0.005.

Based on the TCGA datasets and primary cell lines prepared from the tissue samples of glioblastoma patients, the levels of active STAT3 and stem cell markers were positively correlated with the survival rate. To verify this, we determined the tumorsphere-forming capacity of primary glioblastoma cell lines from GBM12 and GBM14. The increase in the size of tumorsphere was of greater magnitude in GBM14 cells than GBM12 cells (Figure 5A), and in GBM14 cells was decreased by the silencing of STAT3 (Figure 5B). Patient-derived GSC lines have been established from patients with high-grade glioma [28]. The levels of active STAT3 and total STAT3 were highly upregulated in GSCs, particularly in GSC84 and GSC528 cells, compared to GSC19 cells (Figure S3A). ODZ10117 decreased the level of active STAT3 in the cell lines in a concentration-dependent manner (Figure 5C), with comparable or greater efficacy than S3I-201, STA-21, nifuroxazide, napabucasin, and AG-490 in GSC84 and 528 cells (Figure 5D and Figure S3B).

The capability of self-renewal is a critical property of CSCs, and the properties of CSCs are mainly determined by their ability to form tumorspheres and by their expression of stem cell markers. According to the tumorsphere forming ability in vitro, ODZ10117 reduced the ability of GSC84 and GSC528 cells with comparable to greater efficacy than the known STAT3 inhibitors (Figure 5E,F and Figure S3C,D). ODZ10117 decreased the mRNA and protein levels of CSC markers such as NESTIN, SOX2, NANOG, OCT4, and CD133 in GSC84 and GSC528 cells (Figure 5D,G and Figure S3B,E), and reduced the population of CD133-positive cells in GSC528 cells (Figure 5H). In addition, ODZ10117 decreased the viability of both GSCs in a concentration-dependent manner (Figure 5I and Figure S3F). These results indicate that the STAT3 activity and stem cell properties of GSCs are closely correlated, and ODZ10117 is a potent inhibitor of STAT3 and stemness in glioblastoma and GSCs.

### 3.6. ODZ10117 Reduces Tumor Growth in Glioblastoma Xenografts

**Figure 6:**
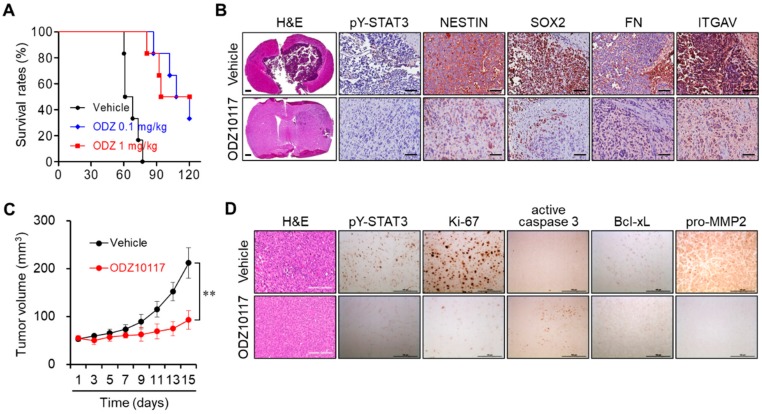
ODZ10117 reduces tumor growth and increases survival in glioblastoma xenograft models. (**A**,**B**) Glioblastoma orthotopic xenograft model was established by injecting GSC528 cells into the right striatum of 6-week-old BALB/c nu/nu nude mice. Kaplan-Meier survival curves of tumor-bearing mice treated with vehicle alone or ODZ10117 (0.1 or 1 mg/kg, *n* = 6) (**A**). H&E and IHC staining of mouse tumor tissues were performed on day 77 after transplantation (**B**). Scale bar: 200 (IHC) or 500 μm (H&E). (**C**,**D**) Subcutaneous tumor xenograft model was established by injecting U87-MG cells suspended in 25% Matrigel in PBS into the flank of BALB/c nude mice (*n* = 6). The mice were treated with vehicle (0.1% DMSO) alone or ODZ1017 (40 μM) at 2-day intervals for 2 weeks. Tumor growth was measured every other day (**C**) and tumor sections were subjected to H&E and IHC staining (**D**). ** *p* < 0.005. Scale bar: 100 μm.

Our results suggest that STAT3 targeting may be a promising therapeutic strategy in patients with glioblastoma. Therefore, we further investigated whether ODZ10117 could suppress tumor growth in in vivo glioblastoma xenograft models. To determine the in vivo pharmacological activity, we generated an orthotopic glioblastoma xenograft model by intracranial injection of GSC528 cells into nude mice. We treated the tumor-bearing mice with vehicle (DMSO:corn oil, 1:9) alone or ODZ10117 (0.1 or 1 mg/kg, daily, intraperitoneal injection) 2 weeks later. ODZ10117 administration increased the median survival rate from 64.5 to 107.0 and 114.0 days, respectively (Figure 6A). When the vehicle-treated control mice exhibited neurological symptoms, the vehicle- and ODZ10117-treated mice were simultaneously euthanized and their tumor tissues were compared. The administration of ODZ10117 dramatically decreased the tumor population and the levels of active STAT3, CSC markers such as NESTIN and SOX2, and MES-associated genes FN and ITGAV without significantly affecting the body weight compared to the vehicle-treated control group (Figure 6B and Figure S4).

We also generated another glioblastoma xenograft model by subcutaneous injection of U87-MG cell suspensions in 25% Matrigel-containing PBS into the dorsal flank of Balb/c nude mice. Vehicle containing 0.1% DMSO or 40 μM ODZ10117 was intratumorally injected into the tumor-bearing mice with a 2-day interval for 2 weeks after 4 weeks of implantation. The tumor growth was dramatically increased in vehicle-treated control mice after 2-weeks, whereas the administration of ODZ10117 suppressed tumor growth (Figure 6C). Upon histological evaluation, the tumor population and the levels of active STAT3, Ki-67, Bcl-xL, and pro-MMP-2 were elevated, and that of active caspase-3 was decreased in vehicle-treated control mice, indicating that the proliferation and growth of tumor cells were active in this group. However, the administration of ODZ10117 suppressed the tumor population and levels of active STAT3 and tumor growth-associated factors and increased the level of active caspase-3 (Figure 6D), indicating the effective anti-tumor effect of ODZ10117. These results indicate that ODZ10117 suppressed tumor growth and GSC maintenance in glioblastoma xenograft models, which increased the survival rate of the mice, suggesting ODZ10117 to be a promising therapeutic candidate for glioblastoma.

## 4. Discussion

Glioblastoma is a grade IV astrocytoma with very poor prognosis, according to the World Health Organization (WHO) [29]. It is the most aggressive and common primary adult brain tumor with a variety of characteristics featured by morphological and genetic heterogeneity [30]. Although there have been extensive advances in studies during the past decades, glioblastoma remains one of the most difficult types of cancers to treat because the exact causes are not clear and therapeutic strategies are limited. In addition, recurrence and resistance to conventional therapies occurs. Therefore, it is necessary to conduct extensive studies to identify the causes and develop novel therapeutic strategies for glioblastoma treatment. STAT3 is one of the key target molecules to treat various types of cancers, including glioblastoma.

Our previous study has shown that ODZ10117 is a novel small molecule compound of STAT3 inhibitor [15]. In this study, we further demonstrated the valuable therapeutic efficacy of ODZ10117 in both in vitro and in vivo models of glioblastoma and GSCs. According to recent studies, aberrantly activated STAT3 signaling is considered a paradigm for tumor initiation and malignancy, radiochemoresistance, and recurrence due to observation in many types of cancers [4,11,31]. These correlations are mainly related to the functions of STAT3 in promoting the migration and invasion and maintaining stem cell properties of cancer cells, which indicates that STAT3 is an attractive molecular target for cancer therapy. In fact, many compounds have been developed to inhibit STAT3 activity directly or indirectly [12,13,14]. In the present study, we observed that the level of active STAT3 was relatively higher in primary cell lines prepared from the tissue samples of glioblastoma patients, and the level was concurrent with the survival rate of the patients. Treatment with ODZ10117 effectively suppressed the tyrosine phosphorylation and nuclear translocation of STAT3, resulting in the effective inhibition of migration, invasion, stem cell properties, and MES signatures in glioblastoma cells and GSCs. In addition, ODZ10117 decreased cell viability and induced apoptotic cell death without affecting the proliferation of glioblastoma cells. Finally, the administration of ODZ10117 markedly inhibited tumor growth established in in vivo mouse xenograft models of glioblastoma and GSCs.

Many types of cancer cells contain a small population of CSCs that possess the characteristics of normal stem cells such as self-renewal, proliferation, and multi-lineage differentiation. CSCs are highly tumorigenic and more resistant to chemotherapy and radiotherapy than non-stem cancer cells [32,33]. Glioblastoma also contains GSCs, which are the major cause of radiochemoresistance and recurrence following conventional therapy and shorter survival [34,35,36,37], suggesting that developing therapeutics targeting GSCs is a novel alternative to treating refractory glioblastoma. According to the TCGA database, primary cell lines established from glioblastoma patients and GSCs, STAT3 is highly activated and its activation, tumor grade and patient survival rate are positively correlated. In addition, STAT3 plays an important role in the generation and maintenance of GSCs and is a key transcription factor leading to the generation of the MES subtype of GSCs [38]. Therefore, it strongly suggests that STAT3 inhibitors may be a valuable strategy for treating glioblastoma by eliminating GSCs. We demonstrated that the expression level of STAT3 and CSCs markers are positively correlated with shorter survival from the TCGA datasets, and ODZ10117 effectively eliminated GSC properties.

**Figure 7:**
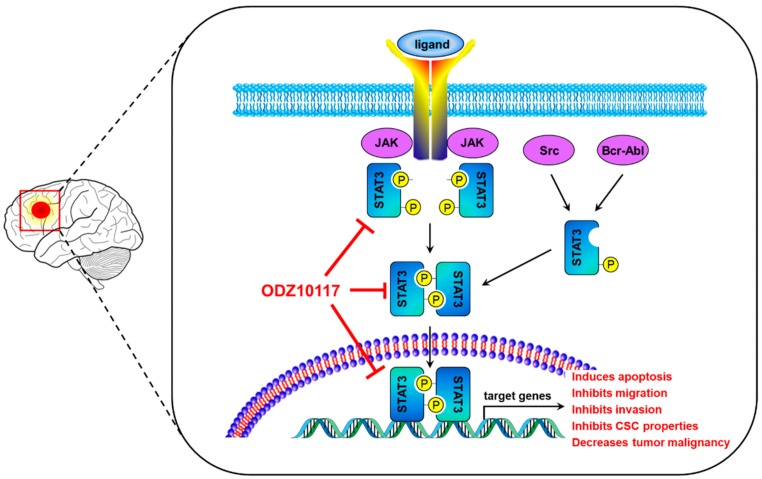
A schematic diagram illustrating the proposed action mechanism of ODZ10117. ODZ10117 specifically targets the activation of STAT3, leading to inhibiting the dimerization, nuclear translocation and DNA binding of STAT3, thereby inducing apoptotic cell death and reducing migration, invasion, cancer stem cell properties, and tumor growth, ultimately inhibiting glioblastoma malignancy.

In conclusion, the present study has shown that ODZ10117 may be a useful candidate for the STAT3-targeted cancer therapy in glioblastoma. ODZ10117 effectively inhibited tyrosine phosphorylation and nuclear translocation of STAT3, resulting in effective anti-tumor activity in both in vitro and in vivo xenograft models of glioblastoma and GSCs. The activities included the suppression of migration, invasion, viability, stem cell properties, and tumor growth, induction of apoptotic cell death, and extension of survival rate by targeting STAT3 in glioblastoma cells, GSCs and glioblastoma xenografts (Figure 7).

## Supplementary Materials

The following are available online at https://www.mdpi.com/2073-4409/9/3/722/s1, Figure S1: ODZ10117 inhibits tyrosine phosphorylation of STAT3 in glioblastoma cells, Figure S2: Positive correlation between STAT3 and stem cell phenotypes and EMT markers with survival in glioblastoma patients, Figure S3: ODZ10117 suppresses stemness features in GSCs, Figure S4: ODZ10117 reduces tumor growth in a glioblastoma xenograft model.

## Abbreviations

CSC	cancer stem cell
DAPI	4′,6-diamidino-2-phenylindole
FITC	fluorescein isothiocyanate
GSC	glioma stem cell
H&E	hematoxylin and eosin
IL-6	interleukin 6
JAK	Janus kinase
PE	phycoerythrin
PI	propidium iodide
qPCR	quantitative real-time polymerase chain reaction
STAT3	signal transducer and activator of transcription 3
TCGA	The Cancer Genomic Atlas
WHO	World Health Organization
