# Supplementary material for: STAT3 Inhibitor ODZ10117 Suppresses Glioblastoma Malignancy and Prolongs Survival in a Glioblastoma Xenograft Model
Source: Cells. 2020 Mar 15;9(3):722. doi: 10.3390/cells9030722 (PMC7140655; doi:10.3390/cells9030722)
Supplement: Supplementary file 1 [file cells-09-00722-s001.pdf]

STAT3 Inhibitor ODZ10117 Suppresses Glioblastoma Malignancy and Prolongs Survival in a Glioblastoma Xenograft Model

Byung-Hak Kim[1,2,3,†], Haeri Lee[1,2,†], Cheol Gyu Park[4,†], Ae Jin Jeong[1,2], Song-Hee Lee[1,2], Kum Hee Noh[1,2], Jong Bae Park[5], Chung-Gi Lee[3], Sun Ha Paek[6,7,8], Hyunggee Kim[4] and Sang-Kyu Ye[1,2,8,9,*]

[1]Department of Pharmacology and [2]Biomedical Science Project (BK21[PLUS]), Seoul National University College of Medicine, Seoul 03080, Republic of Korea

[3]CYTUS H&B Corporation, Cheongju 28159, Republic of Korea

[4]Department of Biotechnology, School of Life Sciences and Biotechnology, Korea University, Seoul 02841, Republic of Korea

[5]Department of System Cancer Science, Graduate School of Cancer Science and Policy, National Cancer Center, Goyang 10408, Republic of Korea

[6]Department of Neurosurgery, [7]Cancer Research Institute, and [8]Ischemic/Hypoxic Disease Institute, Seoul National University College of Medicine, Seoul 03080, Republic of Korea

[9]Neuro-Immune Information Storage Network Research Center, Seoul National University College of Medicine, Seoul 03080, Republic of Korea

***Correspondence:** sangkyu@snu.ac.kr; Tel.: +82-2-740-8281; Fax: +82-2-745-7996

[†]These authors contributed equally to this work.

Supplementary Figures.

Supplementary Figure S1.

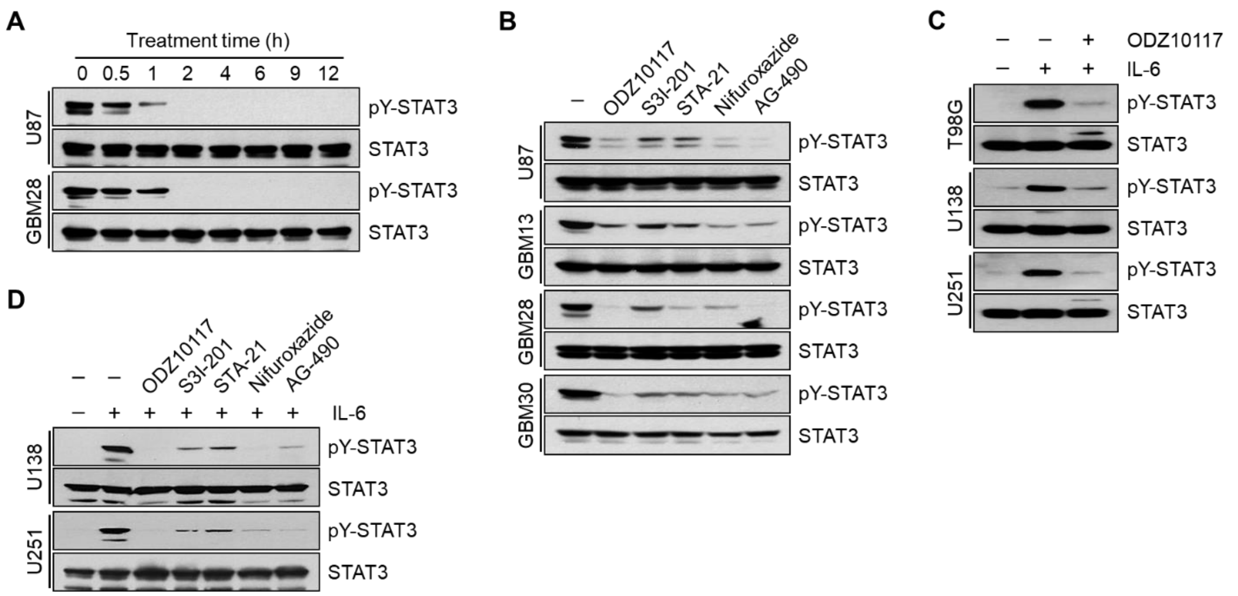

Supplementary Figure S1. ODZ10117 inhibits tyrosine phosphorylation of STAT3 in glioblastoma cells. (A) Cells were incubated with 40 μM ODZ10117 in a time-dependent manner and subjected to Western blot analysis. (B-D) Western blot analysis was performed in cells incubated for 12 h with vehicle (0.1% DMSO) alone, ODZ10117 (40 μM), or the known STAT3 inhibitors S3I-201 (100 μM), STA-21 (100 μM), nifuroxazide (100 μM), and AG-490 (150 μM). Cells were stimulated with IL-6 (20 ng/mL) for 10 min.

Supplementary Figure S2.

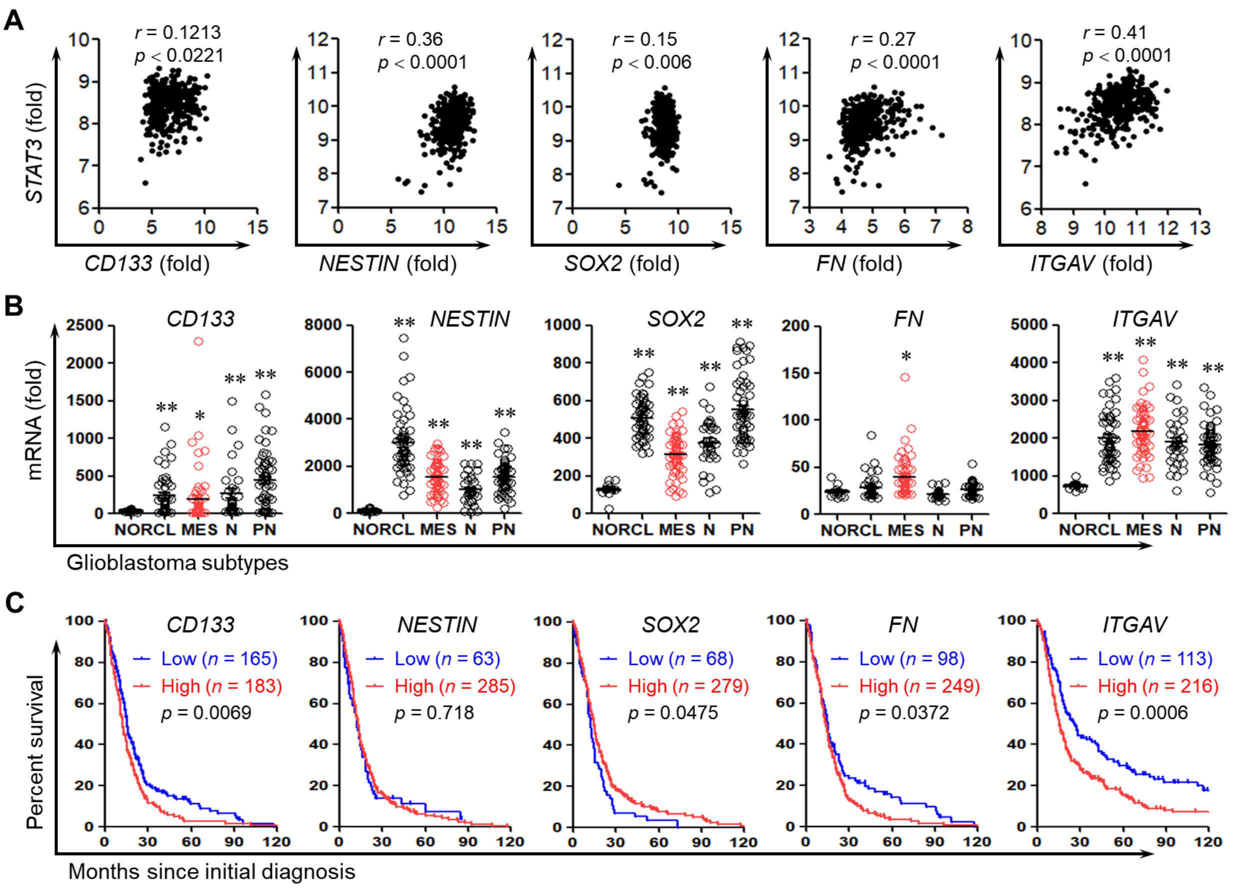

Supplementary Figure S2. Positive correlation between STAT3 and stem cell phenotypes and EMT markers with survival in glioblastoma patients. (A) Positive correlations of the mRNA levels between STAT3 and CD133, NESTIN, SOX2, FN, and ITGAV. (B) The mRNA levels of CD133, NESTIN, SOX2, FN, and ITGAV are elevated in different subtypes of glioblastoma patients. *$p < 0.05$ and **$p < 0.005$. (C) Kaplan–Meier survival curves for glioblastoma patients with CD133, NESTIN, SOX2, FN, and ITGAV. Data from the TCGA database.

Supplementary Figure S3.

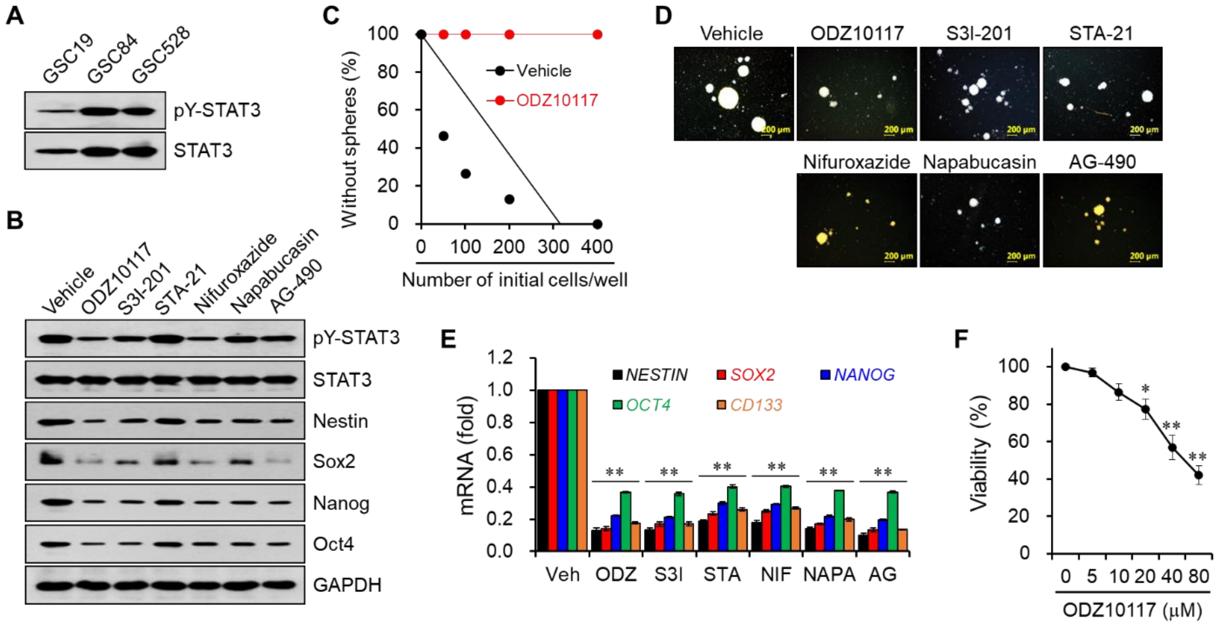

Supplementary Figure S3. ODZ10117 suppresses stemness features in GSCs. (A) The levels of pY-STAT3 and STAT3 in GSCs were determined by Western blot analysis. (B) Comparison of the effect of ODZ10117 and the known STAT3 inhibitors on STAT3 activation and the expression of stem-cell markers in GSC84 cells. GAPDH served as the loading control. (C,D) GSC84 cells were incubated for 5 days with vehicle (0.1% DMSO), ODZ10117 and the known STAT3 inhibitors and *in vitro* limiting dilution (C, $n = 12$) and sphere-forming (D) assays were performed. (E) GSC84 cells were incubated for 24 h with vehicle (0.1% DMSO) alone, ODZ10117 and the known STAT3 inhibitors and the mRNA levels of stem-cell markers were determined by qPCR. ODZ10117 (ODZ, 40 μM), S3I-201 (S3I, 100 μM), STA-21 (STA, 100 μM), nifuroxazide (NIF, 100 μM), napabucasin (NAPA, 4 μM), and AG-490 (AG, 150 μM). (F) GSC84 cells were incubated for 24 h with various concentrations of ODZ10117 and their viability was assayed using the EZ-CyTox Enhanced Cell Viability Assay Reagent. Results are means ± SEM of three independent experiments. *$p < 0.05$ ** and $p < 0.005$.

Supplementary Figure S4.

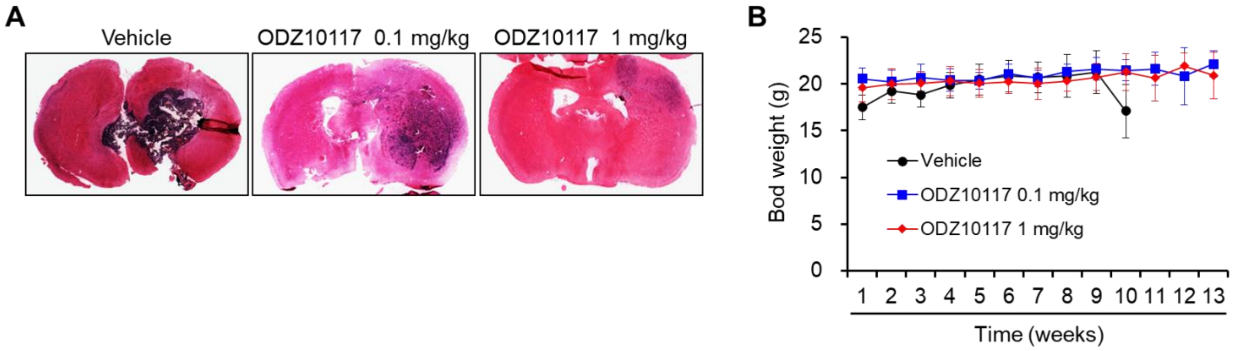

Supplementary Figure S4. ODZ10117 reduces tumor growth in a glioblastoma xenograft model. (A) A glioblastoma orthotopic xenograft model was established by injecting GSC528 cells into the right striatum of 6-week-old BALB/c nu/nu nude mice (*n* = 6). H&E staining of mouse tumor tissues treated with vehicle alone or ODZ10117 (0.1 or 1 mg/kg). (B) Body weight of the tumor-bearing mice were determined.
